# School Performance: A Matter of Health or Socio-Economic Background? Findings from the PIAMA Birth Cohort Study

**DOI:** 10.1371/journal.pone.0134780

**Published:** 2015-08-06

**Authors:** Annemarie Ruijsbroek, Alet H. Wijga, Ulrike Gehring, Marjan Kerkhof, Mariël Droomers

**Affiliations:** 1 Centre for Nutrition, Prevention and Health Services, National Institute for Public Health and the Environment (RIVM), Bilthoven, The Netherlands; 2 Institute for Risk Assessment Sciences (IRAS), University of Utrecht, Utrecht, The Netherlands; 3 Department of Epidemiology and Bioinformatics, University of Groningen, Groningen, the Netherlands; 4 Department of Public Health, Academic Medical Centre, University of Amsterdam, Amsterdam, The Netherlands; San Francisco, UNITED STATES

## Abstract

**Background:**

Performance in primary school is a determinant of children’s educational attainment and their socio-economic position and health inequalities in adulthood. We examined the relationship between five common childhood health conditions (asthma symptoms, eczema, general health, frequent respiratory infections, and overweight), health related school absence and family socio-economic status on children’s school performance.

**Methods:**

We used data from 1,865 children in the Dutch PIAMA birth cohort study. School performance was measured as the teacher’s assessment of a suitable secondary school level for the child, and the child’s score on a standardized achievement test (Cito Test). Both school performance indicators were standardised using Z-scores. Childhood health was indicated by eczema, asthma symptoms, general health, frequent respiratory infections, overweight, and health related school absence. Children’s health conditions were reported repeatedly between the age of one to eleven. School absenteeism was reported at age eleven. Highest attained educational level of the mother and father indicated family socio-economic status. We used linear regression models with heteroskedasticity-robust standard errors for our analyses with adjustment for sex of the child.

**Results:**

The health indicators used in our study were not associated with children’s school performance, independently from parental educational level, with the exception of asthma symptoms (-0.03 z-score / -0.04 z-score with Cito Test score after adjusting for respectively maternal and paternal education) and missing more than 5 schooldays due to illness (-0.18 z-score with Cito Test score and -0.17 z-score with school level assessment after adjustment for paternal education). The effect estimates for these health indicators were much smaller though than the effect estimates for parental education, which was strongly associated with children’s school performance.

**Conclusion:**

Children’s school performance was affected only slightly by a number of common childhood health problems, but was strongly associated with parental education.

## Introduction

Educational attainment is a strong determinant of health and life expectancy. In the Netherlands for example, people with a low level of education have a six year shorter life expectancy than highly educated people. For quality adjusted life years the difference is even larger.[1] It seems likely therefore, that improving educational achievement may have, besides many other benefits, a positive impact on health throughout the life course. To improve educational achievement, given a child’s intellectual capacities, it is important to gain insight into specific subgroups of children that would benefit from additional support throughout their school career. It is well known that children from families with low socio-economic status (SES) are less likely to have successful school careers than children from high SES families.[2–6] Variation in school performance between children from different socio-economic family backgrounds has been shown to emerge early in childhood and tends to increase as children grow older.[7] Besides children from low SES families, also children with poor health may be disadvantaged with respect to educational opportunities.[8] However the evidence on the influence of health on educational achievement is still inconclusive [9–14]. Health may be a determinant of educational achievement besides family SES, but in addition, health problems that are more common among poorer children than among their wealthier peers, may as well be more severe, for instance because their health problems are less well managed.[15] This so-called ‘double disadvantage’ may affect the school performance of these children more severely. A better understanding, of the role of health in children’s school career may facilitate the development of interventions that can help to create a breakthrough in the vicious circle of childhood health affecting educational attainment affecting health status later in life.

Many previous studies investigating the association between health and educational achievement have focused on one specific health condition. In the current study, five common childhood health conditions (asthma symptoms, eczema, general health, frequent respiratory infections, overweight) and school absence due to illness are included, making it possible to examine the relation between health and school performance in a more comprehensive way. We also have longitudinal health information, reported repeatedly on several occasions throughout the child’s primary school years, at our disposal. This makes it possible to examine the impact of the burden of disease during the entire primary school period as well as of fluctuations during this period. Furthermore, we included both an objective measure for the children’s’ school performance (a cognitive test) as well as a subjective school level assessment made by the child’s teacher.

The aim of our study is to provide insight into the influence of health on children’s educational attainment at the transition from primary to secondary education, taking into account parental education. Furthermore, we investigate the hypothesis that poor health may be more detrimental for the educational achievement of children from less educated families than for the educational achievement of children from highly educated families.

## Methods

### Ethics statement

The Medical Ethical Committees of the participating institutes (Rotterdam, start project MEC 132.636/1994/39 and 137.326/1994/130; Groningen, start project MEC 94/08/92; Utrecht, start project MEC-TNO judgement 95/50; Utrecht, age 4 years CCMO P000777C; Utrecht, age 8 years CCMO P04.0071C, protocol number 04-101/K; Rotterdam, age 8 years MEC 2004–152; Groningen, age 8 years M 4.019912; Utrecht, age 12 years METC protocol number 07-337/K) approved the study. Parents, carers or guardians gave written informed consent on behalf of all the minors/children involved in the study.

### Data availability statement

The data underlying the findings presented in this paper are available on request. Requests can be submitted to the PIAMA Principal Investigators. Their names and e-mail addresses are listed on the PIAMA website (http://piama.iras.uu.nl/index-en.php#collaboration). The PIAMA data are not freely accessible in the public domain, because this would be in conflict with the agreement between the PIAMA study team and the PIAMA participants. The information participants received at the start of the study (in 1996–1997) included the statement 'the information that we receive from you will only be used for the PIAMA project' and participants gave written informed consent based on this information.

### Study design and population

Data were obtained from the Dutch PIAMA (Prevention and Incidence of Asthma and Mite Allergy) birth cohort. The PIAMA study was designed to investigate the influence of lifestyle and environmental factors on the development of asthma and allergy. Besides asthma and allergy, the study covered a range of other aspects of child health and development, including respiratory infections, overweight and school performance.[16] Pregnant women were recruited from the general population in three different parts of the Netherlands. Their children (n = 3,963) were born in 1996/1997 and have been followed from birth up to their current age of 17 years. Questionnaires were sent to the participating parents during pregnancy, at three months and yearly from 1 to 8 years of age and at 11 years of age. At the age of 11 years, 3541 families (89.4% of baseline population of 3,963) were still in the study and questionnaires were completed by the parents (n = 2,660) as well as the children themselves (n = 2,651). If parents and/or the child had completed the 11-years-questionnaire, the parents (n = 2,706) subsequently received a short additional school questionnaire on the child’s school performance. This school questionnaire was completed for 2,505 children (93% of those who received the questionnaire and 63% of the baseline population). Next we selected the children whose school level advice had been reported and were still in primary school when they or their parents filled out the 11-years-questionnaire, resulting in 1,865 children.

### Measures

#### School performance

Two indicators of school performance were available. First, scores on a standardized school achievement test, the Central Institute for Test Development (Cito) Test, were used.[17] In most Dutch schools, children have to take this test at the end of their primary school education in order to determine which level of secondary education best suits their abilities. The Cito Test score ranges from 501–550 and can be translated into specific levels of secondary education. In our dataset, the scores ranged from 508 to 550, and were approximately normally distributed.

The second indicator used was the teachers’ assessment of the child with regards to a suitable secondary school level for him or her. This school level assessment of the teacher is based on the entire school career, skills and characteristics of the child. The school level assessment is comprised of seven levels ranging from the lowest Dutch secondary educational level to the highest level. In Table 1, the Dutch educational levels and the Cito Test scores are compared with the International Standard Classification of Education, developed by UNESCO.[18]

**Table 1:** Classification of secondary educational level according to Cito Test scores and Dutch secondary educational levels.

Cito Test score	Dutch secondary educational level	ISCED^a^
-	Special education^b^	ISCED 2C / special education
< 523	Vmbo_b/l	ISCED 2C / preparing for labour market
≥ 523 and < 529	Vmbo_k	ISCED 2C / preparing for labour market
≥ 529 and < 533	Vmbo_g/t	ISCED 2B / preparing for vocational education
≥ 533 and < 537	Vmbo/Havo	ISCED 2A / 2B / Combination class of preparing for vocational education and tertiary education (that is senior general education)
≥ 537 and < 541	Havo	ISCED 2A / preparing for tertiary education (that is senior general education)
≥ 541 and < 545	Havo/Vwo(only offered in the first year of secondary education)	ISCED 2A / preparing for tertiary education (combination class of senior general education and pre-university education)
> 545	Vwo	ISCED 2A / preparing for tertiary education (that is pre-university education)

^a^ International Standard Classification of Education by UNESCO (update 1997)

^b^ education lower than the regular school levels.

Many secondary schools use the Cito Test score and the teacher’s school level assessment as an entry requirement. Both indicators are therefore crucial for the school career of children in the Netherlands. To be able to compare the findings for the two school performance indicators, we standardised the indicators using Z-scores. For our study, the school level assessment was available for all 1,865 children in the study population and the Cito Test score was available for 1,531 children.

#### Socio-economic status measure

Education is considered a good indicator of adult socio-economic status in the Netherlands[19] and elsewhere.[20] Accordingly, both maternal and paternal educational level were used as indicators of the socio-economic status (SES) of the family. Information about the highest level of education attained by the parents was collected around the first birthday of their child, and the information was divided into three categories: primary school only or lower secondary or lower vocational education (low); intermediate vocational education or intermediate or higher secondary education (intermediate); and higher vocational education or university (high). Maternal and paternal educational level were moderately correlated (Pearson’s correlation 0.49).

#### Childhood health indicators

The health of the child was reported by the parents repeatedly between the ages of one to eleven years. We used six health indicators: eczema and frequent respiratory infections (nine reports: at age one to eight years and at age eleven), poor general health (four reports: at ages four, six, eight and eleven), asthma symptoms and overweight (seven reports: at age three to eight years and at age eleven, and school absence due to illness (reported at age 11). Asthma (symptoms), eczema, respiratory infections and overweight are health problems with a relatively high prevalence in childhood and constitute a substantial part of the health problems seen in General Practice among the primary school age group. In addition to these specific common childhood health problems, we used two unspecific ‘overall’ indicators of childhood health: the RAND General Health Rating Index[21] and the number of school days missed due to illness. A child was classified as having eczema if an itchy rash had been present at one or more of the specified locations (the folds of the elbows or behind the knees, around the ears or eyes, or at the front of the ankles) during the previous twelve months. This classification was based on the international standardized ISAAC questions.[22] Frequent respiratory infections were defined as the child having suffered from three or more of the following infections during the previous twelve months: bronchitis, pneumonia, middle ear infection, sinusitis, throat infection, flu or a serious cold. Seven questions from on general health and susceptibility to illness were used to define general health. Answer categories were combined into one score between 7 and 32, with the lowest quartile characterizing poor general health. A Dutch version of the ‘RAND General Health Rating Index’ for children was used for the assessment of general health.[21] Children having wheezing, shortness of breath (dyspnoea), or using prescribed inhaled steroids for respiratory or lung problems in the preceding twelve months were categorized as having asthma symptoms. Overweight was defined using parentally reported weight and height information. Parents were asked to report their child’s body weight (in kg) and height (in cm) from the last time the child was weighed and measured by a medical doctor or nurse, if this had been done within the previous three months. If these measures were not available, the parents were asked to weigh and measure their child themselves, without shoes and heavy clothes. Age- and sex-specific IOTF (International Obesity Taskforce) cut-off points [23], were used to classify the children as being overweight or obese. Because of the low prevalence of obesity, obese and overweight children were children were combined into one category and obesity was not analyzed separately.

We composed two variables per health indicator to reflect childhood health during the period studied and used these variables as independent variables in the analyses. First, for each health indicator separately we calculated the number of years that ill health was reported. Second, for each child, a health trajectory describing the development of the health condition over time was composed separately for each health indicator. The health trajectories were defined as persistent health problems, health problems in early childhood only, health problems in late childhood only and no health problems. Children were categorized in the trajectory of ‘persistent health problems’ when the parents reported health problems at least two out of three times in the period between age three to five and at least two out of four times between the ages six to eleven (in case of poor general health at least once between age three to six and at least once between age seven to eleven); children were categorized in the trajectory of ‘health problems in early childhood only’ when the parents reported health problems at least two out of three times in the period between age three to five years (at least once for poor general health between age three to six), but not more than once between the ages six to eight and age eleven (none for poor general health); children were categorized in the trajectory ‘health problems in late childhood only’ when the parents reported health problems not more than once in the period between age three to five years (none for poor general health), and at least two out of four times later in childhood (six to eight years and at eleven years) (at least once for poor general health between age seven to eleven); the trajectory ‘no health problems’ was composed of children whose parents reported health problems not more than once between age three to five years, and not more than once between the ages six to eleven (and never in case of poor general health). The latter trajectory was the reference category in the analyses.

Finally, in the school questionnaire, parents were asked to report the number of days their child had been absent from school due to illness during the current school year. We divided the answers into no school absence, one or two days, three to five days, and more than five days of school absence due to illness.

### Analyses

We used linear regression models for our analyses with adjustment for sex of the child. We used Z-scores for the two outcome measures; Cito Test scores and school level assessment. We used heteroskedasticity-robust standard errors, because the residuals of the outcome measures were not normally distributed and the variance was not homogeneous.

First, the relationship between the number of years of ill health, absenteeism due to illness, and the health trajectories with the school level assessment and the Cito Test score were investigated. Second, the associations of the educational level of the mother and father with the school level assessment and the Cito Test score were investigated (by constructing dummies out of the parental educational attainment question).

In the next step, both the health status of the children and parental educational level were entered together as independent variables in the regression models with the school level assessment and the Cito Test score as dependent variables. Finally, for each health indicator we investigated whether the associations of health indicators with school performance varied by socio-economic background by including an interaction between parental educational level and health in the models. For significant interactions (P<0.1), [24] we show results of the analyses stratified by parental educational level.

#### Missing data and multiple imputation

The health indicators in our study were constructed using 1 to 9 repeated measurements per indicator. However, not every parent answered every question every year, so that for one or more of the indicators one or more of the repeated measurements may be missing. If data are not missing completely at random (MCAR), complete case analysis may lead to biased results [25,26] and will result in inefficient use of the available data. Therefore, to overcome this problem, missing data in independent variables were imputed multiple times using the ‘Multivariate Imputation by Chained Equations’ (MICE) procedure in the statistical program R, version 2.9.1, resulting in five imputed datasets. SAS analyses were performed with PROC MIANALYSE to pool the data. The means and frequencies of the school performance measures and their determinants were compared for the complete and imputed cases (Table 2) and the analyses were conducted in both the imputed dataset and in the subgroup with complete data. Results presented in Tables 3–5 are the results from the analyses in the imputed dataset.

**Table 2:** Characteristics of the study population: original dataset versus imputed dataset.

	Complete cases (n = 412)	Imputed dataset (n = 1,865) ^a^		Complete cases (n = 412)	Imputed dataset (n = 1,865)^a^
	%	%		%	%
Gender			Poor general health		
Male	54.9	50.8	age 4	20.9	24.1
Female	45.2	49.2	age 6	21.1	20.3
			age 8	23.1	23.2
Maternal education			age 11	24.0	21.6
Low	13.6	17.8			
Intermediate	42.5	41.9	Freq. respiratory infections	
High	43.9	40.3	age 1	10.2	12.5
			age 2	13.6	13.2
Paternal education			age 3	7.8	10.6
Low	21.1	20.9	age 4	8.5	9.4
Intermediate	30.1	33.4	age 5	8.7	9.7
High	48.8	45.8	age 6	6.1	7.4
			age 7	4,1	4.3
Eczema			age 8	2.7	4.0
age 1	13.8	14.0	age 11	3.2	3.2
age 2	18.5	17.3			
age 3	17.2	17.3	Overweight		
age 4	17.5	17.9	age 3	6.8	8.2
age 5	17.7	16.0	age 4	7.3	9.7
age 6	15.1	15.6	age 5	9.2	10.4
age 7	15.5	14.8	age 6	9.7	9.9
age 8	15.1	16.3	age 7	9.2	11.7
age 11	13.6	13.6	age 8	10.9	12.0
			age 11	10.2	11.0
Asthma symptoms					
age 3	19.2	21.9	School absence		
age 4	15.8	17.6	No absence	30.8	30.2
age 5	15.3	15.7	1–2 days	32.3	30.1
age 6	14.6	13.1	3–5 days	24.5	25.2
age 7	11.9	11.4	> 5 days	12.4	14.5
age 8	11.9	12.1			
age 11	9.7	11.4			
	Mean (SD)	Mean (SD		Range	Range
Z-score School level assessment	0.0 (1.0)	0.0 (1.0)		-2.6–1.0	-2.1–1.1
Z-score Cito Test score	0.0 (1.0)	0.0 (1.0)		-3.8–1.3	-3.9–1.4

^a^ Data were not imputed for Cito Test Scores (n = 1,531).

**Table 3:** Association of parental education and health indicators with school level assessment (Z-score) (n = 1,865)^a^.

	Crude^b^	Adjusted for maternal education	Adjusted for paternal education	Adjusted for maternal education and all health indicators	Adjusted for paternal education and all health indicators
	β^c^ (95% CI) ^c^	β^c^ (95% CI) ^c^	β^c^ (95% CI) ^c^	β^c^ (95% CI) ^c^	β^c^ (95% CI) ^c^
**Parental education**					
*Maternal education*					
High (reference)	0			0	
Intermediate	**-0.41** (-0.50;-0.32)			**-0.41** (-0.50;-0.32)	
Low	**-0.92** (-1.05;-0.80)			**-0.90** (-1.03;-0.77)	
*Paternal education*					
High (reference)	0				0
Intermediate	**-0.46** (-0.55;-0.36)				**-0.45** (-0.54;-0.35)
Low	**-0.92** (-1.04;-0.80)				**-0.91** (-1.03;-0.79)
**Health indicators**					
Eczema (no. of years)	-0.00 (-0.02;0.02)	-0.01 (-0.02;0.01)	-0.00 (-0.02;0.02)	-0.00 (-0.02;0.02)	0.00 (-0.02;0.02)
Asthma symptoms (no. of years)	-0.02 (-0.05;0.01)	-0.01 (-0.04;0.01)	-0.02 (-0.04;0.01)	-0.00 (-0.03;0.02)	-0.01 (-0.04;0.025)
Poor general health (no. of years)	-0.04 (-0.07;0.00)	-0.03 (-0.07;0.01)	-0.03 (-0.06;0.01)	-0.02 (-0.06;0.02)	-0.01 (-0.05;0.03)
Freq. respiratory infections (no. of years)	-0.03 (-0.07;0.01)	-0.01 (-0.05;0.02)	-0.02 (-0.06;0.02)	0.00 (-0.04;0.04)	-0.00 (-0.04;0.04)
Overweight (no. of years)	**-0.05** (-0.08;-0.01)	-0.03 (-0.06;0.00)	-0.02 (-0.05;0.01)	-0.03 (-0.06;0.00)	-0.02 (-0.05;0.01)
*School absence*					
0 days (reference)	0	0	0	0	0
1–2 days	0.07 (-0.04;0.19)	0.01 (-0.10;0.12)	0.04 (-0.06;0.15)	0.02 (-0.09;0.13)	0.04 (-0.06;0.15)
3–5 days	0.03 (-0.09;0.14)	-0.00 (-0.12;0.11)	-0.00 (-0.11;0.11)	0.01 (-0.11;0.12)	-0.00 (-0.11;0.12)
>5 days	**-0.18** (-0.33;-0.03)	-0.12 (-0.26;0.02)	**-0.17** (-0.31;-0.03)	-0.10 (-0.24;0.05)	**-0.15** (-0.29;-0.00)

^a^ Adjusted for the sex of the child

^b^ crude models, only adjusted for sex of the child

^c^ 95% confidence intervals of β.

**Table 4:** Association of parental education and health indicators with Cito test score (Z-score) (n = 1,531)^a^.

	Crude^b^	Adjusted for maternal education	Adjusted for paternal education	Adjusted for maternal education and all health indicators	Adjusted for paternal education and all health indicators
	β^c^ (95% CI) ^c^	β^c^ (95% CI) ^c^	β^c^ (95% CI) ^c^	β^c^ (95% CI) ^c^	β^c^ (95% CI) ^c^
**Parental education**					
*Maternal education*					
High (reference)	0			0	
Intermediate	**-0.37** (-0.47;-0.27)			**-0.36** (-0.46;-0.26)	
Low	**-0.85** (-0.99;-0.70)			**-0.82** (-0.97;-0.67)	
*Paternal education*	0				0
High (reference)	**-0.40** (-0.50;-0.29)				**0.39** (-0.50;-0.28)
Intermediate	**-0.78** (-0.92;-0.65)				**-0.77** (-0.90;-0.63)
Low					
**Health indicators**					
Eczema (no. of years)	0.00 (-0.02;0.02)	-0.00 (-0.02;0.02)	0.00 (-0.02;0.02)	0.00 (-0.02;0.03)	0.01 (-0.02;0.03)
Asthma symptoms (no. of years)	**-0.04** (-0.07;-0.01)	**-0.03** (-0.06;-0.00)	**-0.04** (-0.07;-0.01)	-0.03 (-0.06;0.01)	-0.03 (-0.07;0.00)
Poor general health (no. of years)	-0.03 (-0.08;0.01)	-0.03 (-0.07:0.01)	-0.03 (-0.07:0.01)	-0.01 (-0.06;0.05)	0.00 (-0.05;0.06)
Freq. respiratory infections (no. of years)	-0.04 (-0.08;0.00)	-0.02 (-0.06;0.02)	-0.03 (-0.07;0.01)	0.00 (-0.04;0.04)	-0.01 (-0.05;0.04)
Overweight (no. of years)	**-0.06** (-0.10;-0.02)	**-0.04** (-0.07;-0.00)	-0.03 (-0.07;0.01)	-0.03 (-0.07;0.00)	-0.02 (-0.06;0.01)
*School absence*					
0 days (reference)	0	0	0	0	0
1–2 days	0.06 (-0.07:0.19)	-0.00 (-0.13;0.12)	0.04 (-0.09;0.16)	-0.00 (-0.13;0.12)	0.03 (-0.09;0.16)
3–5 days	0.05 (-0.08;0.18)	0.03 (-0.10;0.15)	0.02 (-0.11;0.14)	0.04 (-0.09;0.16)	0.02 (-0.10;0.15)
>5 days	**-0.18** (-0.35;-0.01)	-0.14 (-0.30;0.02)	**-0.18** (-0.34;-0.02)	-0.11 (-0.27;0.05)	-0.16 (-0.32;0.01)

^a^ Adjusted for the sex of the child

^b^ crude models, only adjusted for sex of the child

^c^ 95% confidence intervals of β.

**Table 5:** Association between children’s health trajectories and school level assessment (n = 1,865) and Cito Test score (n = 1,531) (Z-scores).

		School level assessment ^a^	Cito Test score ^a^
	%	β^b^ (95% CI) ^b^	β^b^ (95% CI) ^b^
*Eczema*			
none (reference)^c^	79	0	0
early only^d^	6	-0.01 (-0.22;0.19)	0.06 (-0.18;0.29)
late only^e^	7	-0.12 (-0.32;0.08)	-0.02 (-0.24;0.19)
Persistent^f^	9	-0.07 (-0.22;0.08)	-0.04 (-0.21;0.13)
*Asthma symptoms*			
none (reference)^c^	80	0	0
early only^d^	8	0.03 (-0.14;0.19)	-0.01 (-0.19;0.17)
late only^e^	5	-0.16 (-0.39;0.07)	-0.25 (-0.52;0.02)
Persistent^f^	7	-0.17 (-0.35;0.01)	**-0.25 (-0.46;-0.04)**
*Poor general health*			
none (reference)^c^	53	0	0
early only^d^	14	-0.05 (-0.18;0.09)	-0.08 (-0.23;0.08)
late only^e^	14	0.03 (-0.10;0.17)	0.00 (-0.15;0.15)
Persistent^f^	19	**-0.14 (-0.26;-0.01)**	-0.12 (-0.26;0.02)
*Frequent respiratory infections*			
none (reference)^c^	93	0	0
early only^d^	5	-0.15 (-0.38;0.07)	-0.18 (-0.45;0.09)
late only^e^	2	-0.17 (-0.51;0.17)	-0.18 (-0.52;0.16)
Persistent^f^	1	0.01 (-0.38;0.41)	0.25 (-0.14;0.65)
*Overweight*			
none (reference)^c^	86	0	0
early only^d^	3	0.00 (-0.38;0.39)	-0.15 (-0.65;0.35)
late only^e^	8	**-0.34 (-0.54;-0.14)**	-0.23 (-0.48;0.01)
Persistent^f^	4	-0.08 (-0.31;0.14)	-0.21 (-0.48;0.07)

^a^ Adjusted for sex of the child

^b^ 95% confidence intervals of β

^c^ Parents reported health problems not more than once between age three to five years, and not more than once between age six to eleven (never in case of poor general health)

^d^ Parents reported health problems at least two out of three times between age three to five years (poor general health at least once between age three to six) and not more than once between age six to eleven (none for poor general health)

^e^ Parents reported health problems not more than once between age three to five years (none for poor general health), and at least two out of four times between age six to eleven (poor general health at least once between age seven to eleven)

^f^ Parents reported health problems at least two out of three times between age three to five and at least two out of four times between age six to eleven (poor general health at least once between age three to six and at least once between age seven to eleven).

## Results

### Health and school level assessment

School level assessment was lower for children with overweight (-0.05 z-score (95% CI -0.08; -0.01) per year with overweight) and children who were absent from school for more than five days in the current school year (-0.18 z-score (95% CI -0.33; -0.03), Table 3). Eczema, asthma symptoms, frequent respiratory infections were not associated with the children’s school level assessment, while poor general health was borderline significantly associated with a lower school level assessment (-0.04 z-score (95% CI -0.07; 0.00) per year with poor general health.

Assessment of the health trajectories in relation to the school level assessment showed that only children suffering from poor general health persistently throughout their childhood received a significantly lower school level assessment than children with good general health during childhood (-0.14 z-score (95% CI -0.26;-0.01, Table 5). When poor general health was present only early or only later in childhood, there was no association with school level assessment. Children with overweight later in childhood, but not those with early overweight, received a significantly lower school level assessment than children without overweight during childhood (-0.34 z-score (95% CI -0.54;-0.14)).

### Health and Cito Test score

Cito test scores were significantly lower in children with asthma (-0.04 z-score (95% CI -0.07; -0.01) per year with asthma), in children with overweight (-0.06 z-score (95% CI -0.10; -0.02) per year with overweight) and in children who were absent from school for more than five days in the current year (-0.18 z-score (95% CI -0.35;-0.01)) than in children without these health problems or health related school absence, respectively. Eczema and poor general health were not related to children’s Cito Test scores, while frequent respiratory infections was borderline significantly associated with a lower Cito Test score (-0.04 z-score (95% CI -0.08;0.00) (Table 4).

Considering the health trajectories (Table 5), children with persistent asthma symptoms during childhood scored lower on the Cito Test than children without asthma symptoms (-0.25 z-score). None of the other health trajectories were statistically significantly associated with the Cito Test scores (Table 5).

### Parental educational level and school performance

The lower the educational level of the parents, the poorer their children performed at school (Tables 3 and 4). Children of parents with a low education received a school level assessment that was 0.92 z-score lower and a Cito Test score that was 0.78 to 0.85 z-score lower (respectively paternal and maternal educational level) than their peers with highly educated parents. Children with intermediately educated parents received a school level assessment that was 0.41 to 0.46 z-score lower (respectively maternal and paternal educational level) and a Cito Test score that was 0.37 to 0.40 z-score lower (respectively maternal and paternal educational level).

### Health, parental education and school performance

When parental educational level and the health problems were included in the same regression models (Tables 3 and 4), the association between the parental educational level and children’s school performance remained significant and stable. This was also the case when parental education was included in one regression model with all the health indicators together.

Generally, the strength of the associations between the health indicators and school performance attenuated slightly when parental education was added to the regression models. The association between asthma symptoms and Cito Test Score remained stable after inclusion of maternal as well as paternal education. The association between health related school absence of more than five days in the past year and school performance remained stable after adjustment for paternal education, but the point estimate of school absence decreased when maternal education was included. The associations between overweight and school performance attenuated somewhat after inclusion of maternal education and slightly more when paternal education was included in the model (Tables 3 and 4).

### Association between health and school performance by parental education

**Fig 1:**
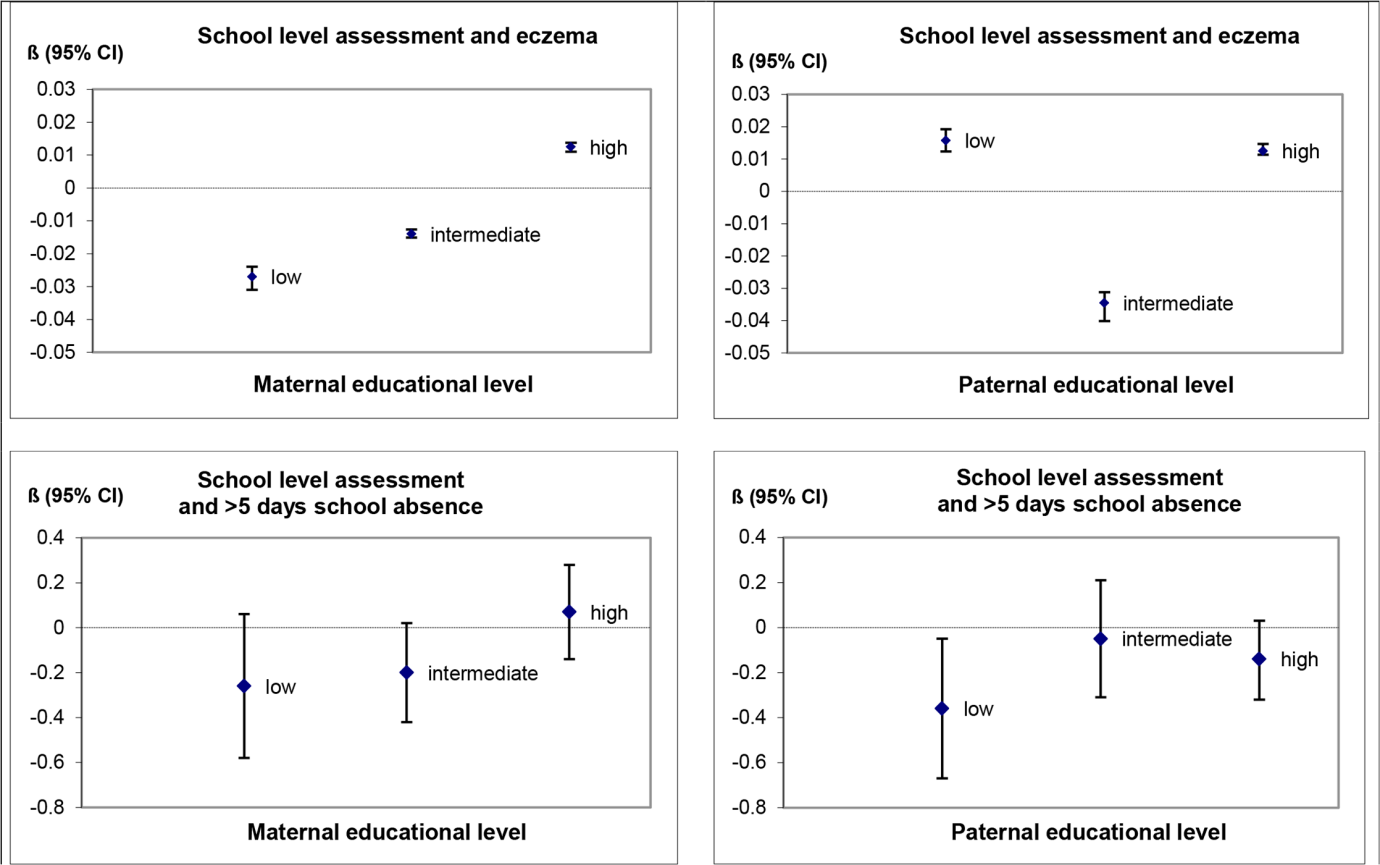
Associations between eczema, health related school absence and school level assessment, stratified by parental education^a^. ^a^ Adjusted for sex of the child.

The associations between the six health indicators and the Cito Test score did not differ by the educational level of the parents. For the school level assessment, we found that maternal education modified the associations of eczema (p for interaction = 0.09), and health related school absence (p-value for interaction = 0.05) with the school level assessment. Fig 1 shows the associations between eczema and health related school absence with the school level assessment, stratified by the educational level of the mother and the father. Associations between eczema and school level assessment differed significantly between different levels of parental education, but the effect estimates were extremely small and the pattern of interaction was inconsistent. Having missed more than 5 schooldays due to illness was associated with a 0.3 z-scores lower school level assessment in children of parents with a low level of education (statistically significant in children with low paternal education). In children of more highly educated parents, the school level assessment was less affected (between -0.2 and 0.1 z-scores) by having missed more than 5 schooldays due to illness.

### Findings from the imputed dataset compared with the complete case dataset

In the complete-case dataset, the prevalence of high parental education was slightly higher and the prevalence of ill health was slightly lower than in the imputed dataset (Table 2). We found that the association between parental education and school performance was smaller in the complete case analyses, though in the same direction (Tables F and G in S1 Table). In the complete case analyses, the association between overweight and children’s school level assessment was larger than in the imputed dataset and remained significant when the educational level of the parents was taken into account. Furthermore, more health problems were associated with the Cito Test score and remained significant when parental educational level was included.

## Discussion

### Main findings

The health problems assessed in our study were not significantly associated with lower school performance, with the exception of asthma symptoms, overweight and missing more than 5 schooldays due to illness. The effect estimates for these health indicators were much smaller though than the effect estimates for parental education, which was strongly associated with children’s school performance. Analysis of the health trajectories showed that for asthma and poor general health, the associations with school performance were significant if these conditions were present persistently throughout the child’s life, but not when they were present in early life only. We found only limited evidence for the hypothesis that poor health would be more detrimental for the school performance of children from less educated parents than for children from highly educated parents. Health related school absence was only associated with children’s school performance if their parents were less educated, but for the other health indicators no such interactions were observed.

### Strengths and limitations

An important strength of our study is the use of data collected prospectively and (with the exception of school days missed) repeatedly, allowing us to make a robust assessment of the health status of children throughout their primary school years and to examine the role of early, late and persistent ill health. Furthermore, this study provided both an objective (Cito Test score) and subjective (teacher’s school level assessment) measure of school performance and information on six different indicators of health.

This study has several limitations. Data of 1,865 children (47% of the baseline study population of 3,963 children) were available for the current study. For 1,531 of these children the Cito Test score was available. Children included in the current study had more highly educated parents than the children from the baseline study population (35% high educated mother and 40% high educated fathers in the baseline study population versus 40% high educated mother and 46% high educated fathers in the current study).[16] However, we see no reason to assume that our results would have been essentially different if the distribution of parental educational levels in our study population would have been more similar to the distribution in the PIAMA baseline population.

As indicators of childhood health, we used a number of specific health problems that are common in primary school children as well as two more general measures of health. Our health measures were not comprehensive however. We did not include very serious but rare diseases and handicaps, nor did we include mental problems and behavioral disorders. Serious diseases and handicaps are relatively rare in the general population and although they are likely to have a strong impact on individual children’s possibilities to attend school, we think it unlikely that not including such diseases has affected the results of our general population study. Mental and behavioral problems, on the other hand, may be more prevalent and may influence school performance in the general population. When interpreting our findings it should be taken into account that mental and behavioral problems were not covered in the study.

We used school absence due to illness as a general measure of the health of a child, but we cannot rule out that other reasons for school absence may have been included in the answers as well, such as a child being reluctant to go to school. This would have resulted in an indicator that not only measures children’s health status, but also their pleasure in going to school, that could also be related to their school performance or academic capacity. However, we believe it is less likely that other reasons for school absence were captured with this measure, because of the specific reference to illness in the question.

We imputed the missing data on the independent indicators multiple times to reduce the risk of selection bias. This also allowed us to make use of the data more efficiently, because all available data were included in the analyses and not only the data of the subgroup that never had a single missing value on the health indicators in the entire study period. In our total dataset only 5.6% of the data were missing, but these missing values were distributed over a large number of participants. If we would use only the complete cases, with no missing data at all, we could only use data of 22% of our study population and we would discard all the available information from 78% of the study population because they had a missing value on one or more of the repeated measurements. For most health conditions, imputation slightly increased the prevalence, indicating that healthy children were somewhat overrepresented in the complete cases. Analyses in the subgroup with complete data showed more significant associations between the health indicators and school performance than we observed in the imputed dataset, indicating that we might have overestimated the effects of health on educational performance if we had restricted our analyses to this subgroup.

### Comparison with other studies

In the current study, lower parental educational level was strongly and significantly associated with lower school performance. This relationship has been found in many studies.[2–6] We investigated whether eczema, asthma symptoms, general health, frequent respiratory infections, overweight and health related school absence were associated with children’s school performance. We found little evidence for a significant association between these health aspects and school performance. In the literature, most studies on health and educational attainment have focussed on asthma, overweight/ obesity or general health.[8] In most cases, these studies addressed only one health condition. Reviews concerning the relation between asthma and school performance reported that the evidence is inconclusive,[8–10] while some individual studies found that (persistent) asthma was associated with worse school performance.[13,14] Reviews concerning overweight and obesity generally conclude that weight problems are associated with worse school achievement.[8,12,27] However, it is not always clear whether the studies described in these reviews adjusted for parental SES. A study by Judge & Jahns (2007) did, and they found that the association between overweight and school performance was no longer statistically significant after adjusting for SES.[10] Case et al. (2005) found that, in a UK cohort of subjects born in 1958, chronic health problems (physical impairments, mental and emotional conditions and other ‘systems’ conditions) during childhood adversely affected children’s educational attainment independent of parental income, education and social class.[28] In accordance with our study though, they reported also that the educational level and social class of the father provided the largest contribution to the educational attainment of the child, and that childhood health contributed much less. Overall, previous studies as well as our study do not provide conclusive evidence that the health conditions studied here have a significant negative effect on school performance independent of socio-economic status of the parents.

Additionally, we examined whether the relationship between health and school performance differed by the educational levels of the parents. The negative impact of health related school absenteeism on the school level assessment was only observed among children with less educated parents, not for their peers with higher educated parents. Our results are in line with the results from studies on asthma by Fowler et al. (1992) and Salm & Schunk (2008), in that they also found that a negative health impact on school performance only occurred among children from low income families [29] or was stronger among children from lower educated parents[30].

### Interpretation and implications of the findings

This study contributes to the discussion on the extent to which health affects education versus education affecting health.[31,32,33] Compared to the associations we observed for the health indicators, the associations between parental education and school performance were much stronger. Effect estimates were fairly similar for paternal and maternal education and showed a clear dose-response relation for the three levels of education. When including the health indicators, the strength of the associations between parental education and school performance was not affected, not even when all health indicators were included together in the models at the same time. Children of parents with a low educational attainment scored, on average, 6 points (absolute, unstandardized points) lower on the CITO test than children of high educated parents. This difference corresponds with a difference of one or two educational levels (out of seven, see Table 1) at the start of their secondary education at the age of 12 years. This will influence their ultimate educational attainment and, eventually, their socio-economic position in adulthood. This is, in turn, an important determinant of health.[34]

Besides heredity of intelligence and cognitive skills,[35,36,37] studies have shown that the availability of a stimulating home environment determines children’s school performance.[36–40] In this context, the educational level of the parents can be considered a proxy for human capital, that is the literacy environment of the home, the parental engagement in school, and the belief in the importance of schooling, which is positively linked to learning abilities in childhood.[39] Governmental initiatives such as “Sure Start’ in the UK and ‘Centre for Youth and Family’ (Centrum voor Jeugd en Gezin) in The Netherlands support parents in creating such stimulating home environments in order to help their children reach their highest (academic) abilities. Our findings underline that proving this type of support is particularly important for parents with a lower educational attainment.

## Supporting Information

S1 Table Results complete case analyses. (DOCX)

## References

[1] Levensverwachting bij geboorte naar opleidingsniveau [Life expectancy at birth by educational level]. Volksgezondheidenzorg.info. 2015. Available: https://www.volksgezondheidenzorg.info/onderwerp/levensverwachting/cijfers-context/bevolkingsgroepen#!node-levensverwachting-bij-geboorte-naar-opleidingsniveau, RIVM: Bilthoven, April 16, 2015.

[2] Bradley RH, Corwyn RF. Socioeconomic Status and Child Development. Ann. Rev. Psychol. 2002; 53: 371–399. 11752490 10.1146/annurev.psych.53.100901.135233

[3] Chevalier A, Harmon C, O’Sullivan V, Walker I. The impact of parental income and education on the schooling of their children. Discussion Paper Series No. 1496. Bonn: The Institute for the Study of Labor (IZA); 2005.

[4] Brooks-Gunn J, Duncan GJ. Effects of poverty on children. The future of children 1997; 7(2):55–71. 9299837

[5] Goodman A, Gisslemann MD, Koupil I. Birth characteristics and early-life social characteristics predict unequal educational outcomes: consistency across Swedish cohorts born 1915–1929 and 1973–180. J Epidemiol Community Health 2010; 64(Suppl): A11.

[6] Dubow EF, Boxer P, Huesmann LR. Long-term Effects of Parents’ Education on Children’s Educational and Occupational Success: Mediation by Family Interactions, Child Aggression, and Teenage Aspirations. Merrill Palmer Q (Wayne State Univ Press). 2009 7 2009; 55(3): 224–249. 10.1353/mpq.0.0030 20390050 PMC2853053

[7] Marmot M, Allen J, Goldblatt P, Boyce T, McNeish D, Grady M, et al Fair Society, healthy lives The Marmot Review. Strategic Review of Health Inequalities in England Post-2010. London: The Marmot Review;2010.

[8] Suhrcke M, de Paz Nieves C. The impact of Health and Health behaviours on educational outcomes in high-income countries: a review of the evidence Copenhagen: WHO Regional Office of Europe; 2011.

[9] Taras H, Potts-Datema W. Childhood Asthma and Student Performance at School. J School Health 2005a; 75(8):296–312. 16179080 10.1111/j.1746-1561.2005.00041.x

[10] Judge S, Jahns L. Association of Overweight with Academic Performance and Social Behavioral Problems: an Update From the Early Childhood Longitudinal Study. J School Health 2007; 77(10):672–678. 18076412 10.1111/j.1746-1561.2007.00250.x

[11] Milton M, Whitehead M, Holland P, Hamilton V. The social and economic consequences of childhood asthma across the lifecourse: a systematic review. Child Care Hlth Dev. 2004; 30(6):711–28. 10.1111/j.1365-2214.2004.00486.x 15527481

[12] Taras H, Potts-Datema W. Obesity and Student Performance at School. J School Health 2005b; 75(8):291–295. 16179079 10.1111/j.1746-1561.2005.00040.x

[13] Liberty KA, Pattemore P, Reid J, Tarren-Sweeny M. Beginning school with asthma independently predicts low achievement in a prospective cohort of children. Chest 2010; 138(6):1349–1355. 10.1378/chest.10-0543 20558555

[14] Moonie S, Sterling DA, Figgs LW, Castro M. The relationship between school absence, academic performance, and asthma status. J School Health 2008; 78(3):140–148. 10.1111/j.1746-1561.2007.00276.x 18307609

[15] Case A, Paxson C. Children’s Health and Social Mobility. In: Future Child, Fall 2006; 16(2):151–173. 10.1353/foc.2006.0014 17036550

[16] Wijga AH, Kerkhof M, Gehring U, De Jongste JC, Postma DS, Aalberse RC, et al Cohort profile: The Prevention and Incidence of Asthma and Mite Allergy (PIAMA) birth cohort. Int J Epidemiol. 2014; 43 (2):527–535. 10.1093/ije/dys231 23315435

[17] Cito. Betekenis van de standaardscore op de Citotoets [Meaning of the standard scores of the Cito Test]. Available: http://www.cito.nl. Accessed 18 January 2010.

[18] ISCED. 1997; Available: http://www.uis.unesco.org/Education/Pages/international-standard-classification-of-education.aspx.

[19] Van Berkel-van Schaik AB, Tax B. Naar een standaardoperationalisatie van sociaal economische status voor epidemiologisch en sociaal-medisch onderzoek [towards a standard operationalisation of socioeconomic status for epidemiological and sociomedical research] Rijswijk, The Netherlands: Ministerie van WCC; 1990.

[20] Oakes JM, Rossi PH. The measurement of SES in health research: current practice and steps towards a new approach. Soc Sci Med. 2003; 56(4):769–784. 12560010 10.1016/s0277-9536(02)00073-4

[21] Post MWM, Kuykenhoven MM, Verheij ThJM, De Melker RA, Hoes AW. De Nederlandse ‘RAND general health rating index for children’: een meetinstrument voor de algemene gezondheid van kinderen. [the Ducth ‘RAND general health rating index for children’: an instrument for the general health of children] Ned Tijdschr Geneeskd. 1998; 142(49):2680–83. 10065224

[22] Asher MI, Keil U, Anderson HR, Beasley R, Crane J, Martinez F, Mitchell EA, et al International Study of Asthma and Allergies in Childhood (ISAAC): rationale and methods. Eur Respir J. 1995; 8: 483–491. 7789502 10.1183/09031936.95.08030483

[23] Cole TJ, Bellizzi MC, Flegal KM, Dietz WH. Establishing a standard definition for child overweight and obesity worldwide: international survey. BMJ 2000; 320:1240 10797032 10.1136/bmj.320.7244.1240 PMC27365

[24] Selvin S. Statistical Analysis of Epidemiologic Data. New York: NY: Oxford University Press; 1999; pp. 213–214.

[25] Klebanoff MA, Cole SR. Use of multiple imputation in the epidemiologic literature. Am J Epidemiol. 2008; 168(4):355–357. 10.1093/aje/kwn071 18591202 PMC2561989

[26] Sterne JAC, White IR, Carlin JB, Spratt M, Royston P, Kenward MG, et al Multiple imputation for missing data in epidemiological and clinical research: potential and pitfalls. BMJ 2009; 338:b2393 10.1136/bmj.b2393 19564179 PMC2714692

[27] Shore SM, Sachs ML, Lidicker JR, Brett NS, Wright AR, Libonati JR. Decreased Scholastic Achievement in Overweight at Middle School Students. In: Obesity 2008; 16(7):1535–1538. 10.1038/oby.2008.254 18451772

[28] Case A, Fertig A, Paxson C. The lasting impact of childhood health and circumstances. J Heal eco. 2005; 24: 365–389. 10.1016/j.jhealeco.2004.09.008 15721050

[29] Fowler MG, Davenport MG, Garg R. School functioning of US children with asthma. Pediatrics 1992; 90(6):939–944. 1437438

[30] Salm M, Schunk D. Child health disparities, socio-economic status, and school enrolment decisions: evidence from German elementary school entrance exams. Adv Health Econ Health Serv Res. 2008; 20:271–288. 19552312

[31] Kawachi I, Adler NE, Dow WH. Money, schooling and health: mechanisms and causal evidence. Ann NY Acad Sci. 2010; 1186: 56–68. 10.1111/j.1749-6632.2009.05340.x 20201868

[32] Bloom DE. Education, health and development Cambridge, MA: American Academy of Arts and Science; 2005 Available: www.amacad.org/publications/ubase_edu_health_dev.pdf (accessed 26 April 2010).

[33] Cohen AK, Syme SL. Education: a missed opportunity for public health intervention? American Journal of Public Health 6 2013, 103(6):997–1001. 10.2105/AJPH.2012.300993 23597373 PMC3698749

[34] Mheen H van de, Stronks K, Bos Je van de, Mackenbach JP. The contribution of childhood environment to the explanation of socio-economic inequalities in health in adult life: a retrospective study. Sco Sci Med. 1997; (44):13–24.

[35] Anger S, Heineck G. Do smart parents raise smart children? The intergenerational transmission of cognitive abilities. J Popul Econ. 2010; 23:1255–1282.

[36] Deary IJ, Johnson W, Houlihan LM. Genetic foundation of human intelligence. Hum Genet. 2009; 126:215–222. 10.1007/s00439-009-0655-4 19294424

[37] Crawford C, Goodman A, Joyce R. Explaining the socio-economic gradient in child outcomes: the inter-generational transmission of cognitive skills. Longitudinal and Life Course Studies 2010; 2(1):77–93.

[38] Martin SL, Ramey CT, Ramey S. The Prevention of Intellectual Impairment in Children of Impoverished Families: Findings of a Randomized Trial of Educational Day Care. Am J Public Health 1990; 80(7): 844–947. 2356909 10.2105/ajph.80.7.844 PMC1404993

[39] Smith J.R., Brooks-Gunn J., Klebanov P.K. Consequences of living in poverty for young children’s cognitive and verbal ability and early school achievement In: Duncan GJ, Brooks-Gunn J, editors. Consequences of growing up poor. New York: Russel Sage Foundation; 1997, pp. 132–189.

[40] Burchinal MR, Peisner-Feinberg E, Pianta R, Howes C. Development of academic skills from preschool through second grade: family and classroom predictors of developmental trajectories. J School Psychol. 2002; 40(5):415–436.

