# Supplementary material for: School Performance: A Matter of Health or Socio-Economic Background? Findings from the PIAMA Birth Cohort Study
Source: PLoS One. 2015 Aug 6;10(8):e0134780. doi: 10.1371/journal.pone.0134780 (PMC4527686; doi:10.1371/journal.pone.0134780)
Supplement: S1 Table — (DOCX) [file pone.0134780.s001.docx]

**S1 Table Results complete case analyses**

**Table F Association of parental education and health indicators with school level assessment (Z-scores) (n=412)**

|  | **Crude**^b^ | **Adjusted for maternal education** | **Adjusted for paternal education** | **Adjusted for maternal education and all health indicators** | **Adjusted for paternal education and all health indicators** |
| --- | --- | --- | --- | --- | --- |
|  | β^c^ (95% CI) ^d^ | β^c^ (95% CI) ^d^ | β^c^ (95% CI) ^d^ | β^c^ (95% CI) ^d^ | β^c^ (95% CI) ^d^ |
| **Parental education** |  |  |  |  |  |
| *Maternal education* |  |  |  |  |  |
| High (reference) | 0 |  |  | 0 |  |
| Intermediate | **-0.32** (-0.49;-0.15) |  |  | **-0.32** (-0.48;-0.15) |  |
| Low | **-0.87** (-1.14;-0.60) |  |  | **-0.82** (-1.09;-0.55) |  |
|  |  |  |  |  |  |
| *Paternal education* |  |  |  |  |  |
| High (reference) | 0 |  |  |  | 0 |
| Intermediate | **-0.33** (-0.51;-0.14) |  |  |  | **-0.29** (-0.47;-0.11) |
| Low | **-0.73** (-0.96;-0.49) |  |  |  | **-0.68** (-0.91;-0.45) |
| **Health indicators** |  |  |  |  |  |
| Eczema (no. of years) | 0.01 (-0.03;0.04) | 0.01 (-0.03;0.04) | 0.01 (-0.03;0.04) | 0.01 (-0.03;0.04) | 0.00 (-0.03;0.04) |
| Asthma symptoms (no. of years) | -0.01 (-0.06;0.04) | -0.02 (-0.07;0.02) | -0.01 (-0.06;0.04) | 0.01 (-0.04;0.07) | 0.03 (-0.02;0.08) |
| Poor general health (no. of years) | -0.06 (-0.14;0.01) | -0.07 (-0.14;0.01) | -0.06 (-0.13;0.02) | -0.05 (-0.14;0.04) | -0.05 (-0.14;0.04) |
| Freq. respiratory infections (no. of years) | -0.07 (-0.16;0.03) | -0.08 (-0.17;0.01) | -0.07 (-0.16;0.02) | -0.05 (-0.14;0.04) | -0.05 (-0.13;0.04) |
| Overweight (no. of years) | **-0.13** (-0.20;-0.06) | **-0.11** (-0.18;-0.04) | **-0.10** (-0.17;-0.03) | **-0.11** (-0.18;-0.04) | **-0.10** (-0.17;-0.03) |
| *School absence* |  |  |  |  |  |
| 0 days (reference) | 0 | 0 | 0 | 0 | 0 |
| 1-2 days | 0.07 (-0.04;0.19) | 0.01 (-0.10;0.12) | 0.04 (-0.06;0.15) | 0.09 (-0.11;0.28) | 0.17 (-0.02;.37) |
| 3-5 days | 0.03 (-0.09;0.15) | -0.00 (-0.11;0.11) | -0.00 (-0.12;0.11) | 0.06 (-0.15;0.27) | 0.03 (-0.19;0.24) |
| >5 days | **-0.18** (-0.33;-0.03) | -0.13 (-0.27;0.02) | **-0.17** (-0.31;-0.03) | -0.04 (-0.33;0.26) | 0.01 (-0.28;0.30) |

^a^ Adjusted for the sex of the child

^b^ crude models, only adjusted for sex of the child

^c^ Difference in mean school level assessment or Cito Test score with the reference category, for school absence the difference in mean school level assessment or Cito Test score for each day of school absence due to illness

^d^ 95% confidence intervals of β

**Table G Association of parental education and health indicators with Cito test score (Z-scores) (n=412)**

|  | **Crude**^b^ | **Adjusted for maternal education** | **Adjusted for paternal education** | **Adjusted for maternal education and all health indicators** | **Adjusted for paternal education and all health indicators** |
| --- | --- | --- | --- | --- | --- |
|  | β^c^ (95% CI) ^d^ | β^c^ (95% CI) ^d^ | β^c^ (95% CI) ^d^ | β^c^ (95% CI) ^d^ | β^c^ (95% CI) ^d^ |
| **Parental education** |  |  |  |  |  |
| *Maternal education* |  |  |  |  |  |
| High (reference) | 0 |  |  | 0 |  |
| Intermediate | **-0.30** (-0.49;-0.11) |  |  | **-0.30** (-0.48;-0.12) |  |
| Low | **-0.86** (-1.17;-0.54) |  |  | **-0.82** (-1.13;-0.51) |  |
|  |  |  |  |  |  |
| *Paternal education* |  |  |  |  |  |
| High (reference) | 0 |  |  |  | 0 |
| Intermediate | **-0.32** (-0.52;-0.11) |  |  |  | **-0.27** (-0.48;-0.07) |
| Low | **-0.75** (-1.01;-0.49) |  |  |  | **-0.70** (-0.95;-0.44) |
| **Health indicators** |  |  |  |  |  |
| Eczema (no. of years) | 0.00 (-0.03;0.04) | 0.00 (-0.03;0.04) | 0.01 (-0.03;0.04) | 0.01 (-0.03;0.06) | 0.01 (-0.03;0.05) |
| Asthma symptoms (no. of years) | -0.04 (-0.09;0.02) | -0.05 (-0.10; 0.00) | -0.04 (-0.09;0.01) | -0.01 (-0.07;0.05) | 0.01 (-0.06;0.07) |
| Poor general health (no. of years) | **-0.09** (-0.18;-0.00) | **-0.09** (-0.18:-0.01) | **-0.09** (-0.17:-0.00) | -0.05 (-0.15;0.06) | -0.05 (-0.15;0.05) |
| Freq. respiratory infections (no. of years) | -0.11 (-0.23;0.00) | **-0.13** (-0.24;-0.01) | **-0.11** (-0.22;-0.00) | -0.08 (-0.19;0.02) | -0.08 (-0.19;0.02) |
| Overweight (no. of years) | **-0.14** (-0.22;-0.05) | **-0.12** (-0.20;-0.04) | **-0.11** (-0.19;-0.03) | **-0.11**(-0.19;-0.04) | **-0.10**(-0.18;-0.02) |
| *School absence* |  |  |  |  |  |
| 0 days (reference) | 0 | 0 | 0 | 0 | 0 |
| 1-2 days | 0.06 (-0.07:0.19) | -0.01 (-0.13;0.12) | 0.04 (-0.09;0.16) | 0.03 (-0.10;0.25) | 0.12 (-0.10;0.34) |
| 3-5 days | 0.05 (-0.08;0.18) | 0.03 (-0.10;0.15) | 0.02 (-0.11;0.14) | 0.05 (-0.18;0.28) | 0.02 (-0.21;0.25) |
| >5 days | **-0.19** (-0.36;-0.02) | -0.15 (-0.31;0.01) | **-0.19** (-0.35;-0.03) | -0.06 (-0.37;0.25) | -0.01 (-0.33;0.31) |

^a^ Adjusted for the sex of the child

^b^ crude models, only adjusted for sex of the child

^c^ Difference in mean school level assessment or Cito Test score with the reference category, for school absence the difference in mean school level assessment or Cito Test score for each day of school absence due to illness

^d^ 95% confidence intervals of β
